# Hybrid tumor ‘spiradenocylindroma’ with unusual dermoscopic features^☆^

**DOI:** 10.1016/j.abd.2021.07.011

**Published:** 2023-01-02

**Authors:** Ecem Bostan, Etkin Boynuyogun, Ozay Gokoz, Ibrahim Vargel

**Affiliations:** a Cihanbeyli Public Hospital, Konya, Turkey; b Department of Plastic and Reconstructive Surgery, Hacettepe University, Faculty of Medicine, Ankara, Turkey; c Department of Pathology, Hacettepe University, Faculty of Medicine, Ankara, Turkey

Dear Editor,

Spiradenocylindroma is an adnexal neoplasm that shows histopathological characteristics of both spiradenoma and cylindroma.^1^ Cylindromas and the hybrid tumor ‘spiradenocylindromas’ are most commonly located on the face and scalp.^1^ Dermoscopy may provide significant clues for the diagnosis of adnexal tumors. Herein, we would like to report a rare case of spiradenocylindroma with distinctive dermoscopic features.

A 78-year-old woman with a history of hypertension was appointed to our clinic with the complaint of a nodule on the right forehead present for eight years. A detailed history taken from the patient revealed that the nodule was excised seven years ago but recurred within the last year. Dermatologic examination revealed a pinkish, shiny, telangiectatic nodule on the right forehead (Fig. 1). In order to aid with the diagnosis, a dermoscopy was performed which revealed thick arborizing vessels, a blue-white veil, and shiny white streaks and dots/clods resembling rosettes on a violaceous-milky red background (Fig. 1). Our differential diagnoses were nodular basal cell carcinoma, pilomatrixoma, sebaceous carcinoma and dermatofibrosarcoma protuberans. A punch biopsy was performed from the nodule and showed lobules surrounded by and containing eosinophilic basement membrane-like material. The islands were formed by two types of cells of which the peripheral ones are darker with less cytoplasm and the central ones are paler with vesicular nuclei. Spiradenomatous parts were characterized by small basaloid cells with intraepithelial lymphocytes and lymphatic like material in the stroma The two types of elements were intermingled (Figure 2, Figure 3). Immunohistochemical staining features are shown in Fig. 4. The final diagnosis was spiradenocylindroma and total excision was performed.

**Figure 1:**
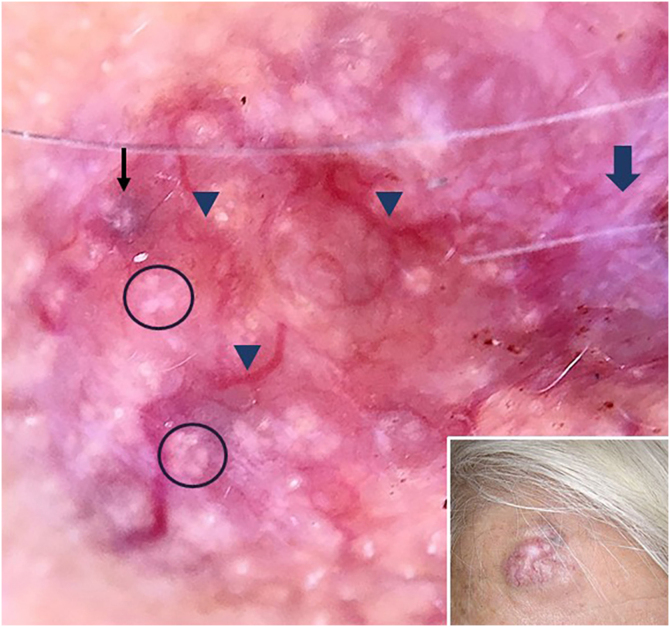
Pink shiny nodule on the right forehead (inset), polarized dermoscopy showed blue/gray-white veil (thin black arrow), bright-white clods and dots resembling rosettes (circles), arborizing vessels (arrowheads) and white shiny streaks (thick arrow).

**Figure 2:**
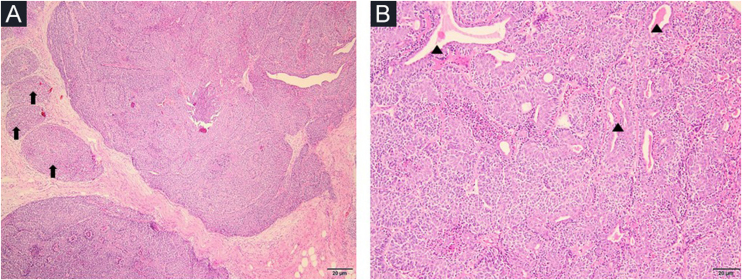
Nodules of different sizes (arrows); larger ones representing mostly the spiradenomatous component (A) (Hematoxylin & eosin, ×40). Bilayered ductal elements within solid aggregations, intraluminal secretory material (arrowheads) (B) (Hematoxylin & eosin, ×100).

**Figure 3:**
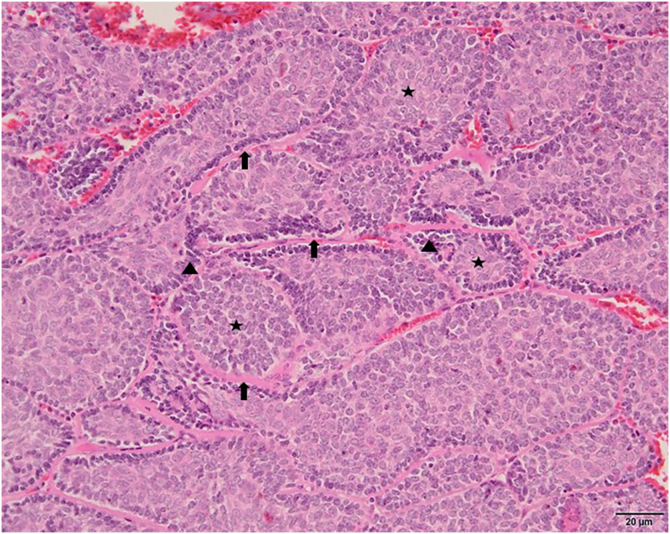
Jigsaw puzzle-like arrangement of islands of cells surrounded incompletely by eosinophilic basement membrane-like material (arrows). Peripheral basophilic palisading cells (arrowheads) with centrally located paler elements (asterisks) (Hematoxylin & eosin, ×200).

**Figure 4:**
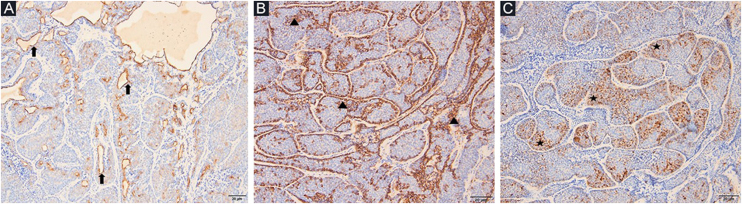
CK5/6 positivity in areas with ductal differentiation (arrows) (A) (CK5/6 ×100). Peripheral layer of myoepithelial cells (arrowheads) (B) (smooth muscle actin ×100). Intratumoral dendritic cells (asteriks) (C) (S100 ×100).

Spiradenocylindromas are benign cutaneous adnexal neoplasms that are derived from apocrine or eccrine glands, demonstrating histopathological features of both spiradenoma and cylindroma.^1^ Since a variety of benign and malignant skin disorders such as basal cell carcinoma, dermatofibrosarcoma protuberans, trichoepithelioma, and microcystic adnexal carcinoma may be considered in the differential diagnoses, histopathological examination remains the gold standard. Spiradenocylindromas are characterized by basaloid cells with eosinophilic basement membrane and tubular structures forming a multinodular pattern as the spiradenomatous part; whereas the cylindromatous portion is mainly composed of small nests of triangular or polyhedral tumoral cells forming a complex pattern resembling a jigsaw puzzle.^2^ Dermoscopy may provide helpful clues to aid in the diagnosis. Senarega et al.^3^ reported a case of spiradenocylindroma which showed linear vessels on a pinkish accompanied by homogeneous blue pigmentation at the periphery. Interestingly, our case showed shiny white streaks along with closely aggregated bright white clods resembling four-dots, five-dots, or cross-like rosettes.^4^ Even though rosettes are most characteristically associated with actinic keratosis, and squamous cell carcinoma; they may also be seen in basal cell carcinoma, melanocytic nevus and dermatofibroma.^5^ Rosettes result from the crossed polarization of horny material in adxenal structures or perifollicular fibrosis.^5^

To our knowledge, our patient is the first spiradenocylindroma case that shows rosette-like structures dermoscopically. By defining dermoscopic features of cylindroma, we want to emphasize the fact that spiradenocylindromas can demonstrate dermoscopic findings such as blue-gray clods and rosette-like structures which may also be detected in other cutaneous neoplasms leading to diagnostic confusion.^5^

## Financial support

None declared.

## Authors’ contributions

Ecem Bostan: Preparation and writing of the manuscript; Data collection, analysis and interpretation; Critical literature review.

Etkin Boynuyogun: Data collection; Approval of the final version of the manuscript.

Ozay Gokoz: Data collection, analysis and interpretation; Approval of the final version of the manuscript.

Ibrahim Vargel: Data collection, analysis and interpretation; Approval of the final version of the manuscript.

## Conflicts of interest

None declared.
